# Surveillance for the Management of Small Renal Masses

**DOI:** 10.1155/2008/196701

**Published:** 2008-08-12

**Authors:** Mehmet Ozsoy, Tobias Klatte, Matthias Waldert, Mesut Remzi

**Affiliations:** Department of Urology, Medical University of Vienna, Währinger Gürtel 18-20, 1090 Vienna, Austria

## Abstract

Surveillance is a new management option for small renal masses (SRMs) in aged and infirm patients with short-life expectancy. The current literature on surveillance of SRM contains mostly small, retrospective studies with limited data. Imaging alone is inadequate for suggesting the aggressive potential of SRM for both diagnosis and followup. Current data suggest that a computed tomography (CT) or magnetic resonance imaging (MRI) every 3 months in the 1st year, every 6 months in the next 2 years, and every year thereafter, is appropriate for observation. The authors rather believe in active surveillance with mandatory initial and followup renal tumor biopsies than classical observation. Since not all SRMs are harmless, selection criteria for active surveillance need to be improved. In addition, there is need for larger studies in order to better outline oncological outcome and followup protocols.

## 1. INTRODUCTION

During the last 20 years, the incidence of renal cell cancer (RCC) has been steadily increasing (2-3%/year) [1]. This rise is mostly due to the increase in detection of incidental small renal masses (SRM ≤ 4 cm) by widespread use of cross-sectional imaging techniques such as ultrasonography (USG), computed tomography (CT), and magnetic resonance imaging (MRI). Between 1983 and 2002, the detection of SRM showed almost a three-fold increase for tumors <2 cm and 2–4 cm, respectively, whereas the detection rate of 4–7 cm and >7 cm just rose by 50% and 26%, respectively [2]. Nowadays, more than 50% of all renal tumors are SRM [3]. The majority of these tumors are asymptomatic and carry a good prognosis [4]. For open and laparoscopic nephron-sparing surgery (NSS), 5-year cancer-specific survival rates of 96–100% have been reported [5, 6].

The highest incidence of incidentally detected SRM is seen in elderly patients [4], who usually present with a number of comorbidities [7–9]. To manage these patients, a variety of treatment options have evolved in the past several years ranging from radical nephrectomy to observation for SRM. NSS remains the standard of care for small RCC, but minimal-invasive therapies and surveillance evolved as alternative treatment options. Here, we review the literature on surveillance for the management of SRM.

## 2. ARE SMALL RENAL MASSES ALWAYS MALIGNANT?

Up to 20% of SRM are actually benign [10–12]. Unfortunately, tumor size alone does not appear to be a predictor of benign or malignant tumor biology. Frank et al. retrospectively examined 2935 solid renal tumors at all sizes treated over a 25-year period and reported 46.3%, 22.4%, 22%, and 19.9% of renal lesions lower than 1 cm, 2 cm, 3 cm, and 4 cm to be benign, respectively [12]. In the prospective randomized multicenter EORTC 30904 study, comparing nephron-sparing surgery with nephrectomy in patients with resectable RCC, 11.6% of the 541 surgically removed tumors (≤5 cm) were benign [13] and among the 100 lesions (mean diameter 2.8 cm) on which Gill et al. [14] performed laparoscopic partial nephrectomy 30% were identified as benign. In a recent report by Remzi et al. [11] that reflects the renal tumors of today, renal tumors of ≤2 cm, 2-3 cm, 3-4 cm were reported to be benign in 24.6%, 20.4%, and 16.0%, respectively, without any correlation to tumor size (*P* = .66). In another series of 1208 SRM, the frequency of benign lesions in the tumor size ranges 0.1–1.0, 1.1–2.0, and 2.1–3.0 was 15%, 14%, and 14%, respectively. However, the incidence of benign lesions decreased significantly in tumors measuring 3.1–4.0 cm (8%, *P* = .001) [10].

Benign lesions (oncocytomas, low-fat angiomyolipomas) are still difficult to differentiate from RCC's even with today's advanced imaging modalities. In a recent study by Remzi et al. [3], only 17% of all benign lesions were correctly identified as benign on preoperative CT and 43% of patients who were assessed incorrectly on preoperative CT's underwent unnecessary radical surgery. There are only a few studies pointing out the success of differentiating low-fat AML from RCC with nonroutine special imaging studies [15].

In addition to initial tumor size, growth rate of tumors on surveillance is also not a reliable predictor of histology. In their review, Chawla et al. compared initial tumor size and the observed growth rate between oncocytomas and RCC variants. Of the 76 tumors, 12% were oncocytomas and the remaining 88% were RCC. At first presentation, mean tumor sizes were 2.00 ± 0.99 (median 1.50, ranging from 1 to 3.9) and 2.21 ± 1.5 cm (median 2.0, ranging from 0.20 to 12.0) in oncocytomas and RCC's, respectively, (*P* = .59). The mean growth rate of oncocytomas and RCC variants did not differ statistically (0.05 ± 0.67, median 0.16, ranging from 1.62 to 0.62, and 0.35 ± 0.41 cm yearly, median 0.35, ranging from 0.42 to 1.6, respectively, *P* = .15) [8]. Thus growth rate after opting for surveillance strategy is not an ideal parameter to further initiate surgical treatment [12–14].

## 3. ARE SMALL RENAL CELL CARCINOMAS HARMLESS?

In their study, Remzi et al. [11] retrospectively analyzed 287 SRMs that were defined to be ≤4 cm by preoperative CT scans and subsequently underwent surgery. About 80% of these lesions were malignant. Tumors were stratified into three groups according to their largest diameter, defined as ≤2 cm, 2.1 to 3.0 cm and 3.1 to 4.0 cm. They were also grouped into two groups of ≤3 cm and 3.1 to 4 cm. There was a significant correlation between tumor size and Fuhrman grade. Two (4.2%), four (5%), and twenty five (25.5%) cases of RCC 2 cm or less, 2.1 to 3 cm, and 3.1 to 4 cm in diameter had Fuhrman grade G3/4, respectively, (*P* = .0007). but there was no statistical difference in Fuhrman grades G3/4 between those ≤2 and 2.1 to 3 cm (*P* = .847), whereas the difference between ≤3 cm and 3.1 to 4 was statistically significant (*P* = .0023). Advanced stage (pT3a or greater) was documented in two (4.2%), 12 (14.9%) and 35 (35.7%) cases for RCC diameter ≤2 cm, 2.1 to 3.0 cm and 3.1 to 4 cm, respectively, (*P* = .0023). At least pT3a stage showed no statistical difference between ≤2 cm and 2.1 to 3 cm group (*P* = .172) whereas the difference between ≤3 cm and 3.1 to 4 cm groups was statistically significant (*P* = .0007). Among the 287 patients, 14 present with distant metastases, 10 of which being among the 119 tumors within the 3.1 to 4 cm group (8.4%) and the remaining four among the 168 tumors of ≤3 cm group (2.4%) (*P* = .0045). This study showed a high-aggressive potential of SRM beyond 3 cm, and thus not all SRM are actually harmless [11].

Klatte et al. [10] investigated 1208 patients with SRM, of whom 88% had RCC. Mean tumor size (±SD) was 2.9 (±0.9) cm. In their study, cancer-specific survival of small nonmetastatic (NX/N0M0) RCC was 96% and 91% after 5 and 10 years, respectively. There was a 7% chance of RCC recurrence post nephrectomy at 5 years. Independent prognostic factors of cancer-specific survival were ECOG performance status, T stage, presence of metastatic disease, and Fuhrman nuclear grade. This study pointed out that there is a small but not insignificant number of patients who recur after curative surgery for SRM.

Measuring tumor diameters by sequential imaging modalities are also not reliable, so when choosing surveillance as an option the cut-off diameter should be set well. As stated above, SRM with a tumor diameter below 3 cm on CT seems to fit better for surveillance than larger tumors. In addition to size, patients with concomitant invasion of the perirenal fat (clinical T3a) on cross-sectional imaging should be excluded from a surveillance protocol, since T3a tumors are at a higher risk of RCC-specific death [10].

In their study, Minardi et al. warn against the possibility of recurrence and death in patients even with low-grade RCC's. They report on 48 patients with pT1a clear cell RCC who underwent NSS. After a median followup of 2 years, 3.9% had died of metastatic RCC. Thus even small lesions can metastasize [16].

## 4. NEW TREATMENT OPTIONS

Nowadays, NSS is the standard treatment for SRM, which is related to the minimal impairment of renal function and excellent cancer-specific survival rates either in open and laparoscopic NSS of about 96–100% after 5 years [5, 6].

Recently minimal invasive therapy modalities such as radiofrequency ablation (RFA), High-intensity focused ultrasound (HIFU) and cryotherapy emerged as potential treatment options for clinically localized RCC for SRM with promising short-term results [17]. Effective renal cryoablation has been achieved by open and laparoscopic approaches as well as by percutaneous image-guided techniques. Percutaneous RFA has been successfully performed under ultrasound, CT, or MRI guidance [18]. Many studies report excellent cancer-specific survival rates of 90–100% [18, 19], however most of the series do not describe the underlying tumor entity (e.g., benign/malignant), have small number of patients treated and a short followup. Additionally Klingler et al. recently reported that skipping (up to 24%) was a major problem in RFA [20]. It is well known from series of open NSS that the time to recurrence is in mean over 5 years, thus a followup of one or two years, which is reported in most series is insufficient to show oncological safety [5, 21]. In a recent meta-analysis on RFA, cryoablation, and surveillance, the risk of recurrence was 7.45 and 18.23 higher for cryoablation and RFA, compared to NSS. Additionally this meta-analysis showed that NSS, ablation, and surveillance are viable strategies for SRMs based on short-term and intermediate term oncological outcomes [18]. However, a significant selection bias currently exists in the clinical application of these techniques with regard to patient age and tumor size. Although long-term data have demonstrated excellent outcomes for NSS, extended oncological efficacy remains to be established for ablation and surveillance strategies. While current data demonstrate a significantly higher incidence of local tumor progression following cryoablation and RFA, no significant differences in progression to metastatic RCC were seen regardless of treatment modality [18]. These data suggest an overtreatment bias for SRMs, thus nowadays a new treatment strategy is “surveillance: treatment by initial observation with serial imaging with delayed treatment for progression” gains popularity as a management option for SRMs.

## 5. ACTIVE SURVEILLANCE IN WHOM, HOW AND WHEN?

### 5.1. Natural history

The natural history of SRM is not well known due to their early removal after diagnosis in most of the cases. There are only few insights on the natural history of SRMs.

In one of the important studies regarding surveillance Bosniak and colleagues retrospectively examined the followup images of 40 incidentally detected renal masses (<3.5 cm). After an average of 3.8 years, 26 tumors were removed. Of these 26 removed masses, 22 (84.6%) were histologically confirmed as RCC. The overall mean linear growth rate for all tumors was 0.36 cm per year. None of the patients developed metastasis [22].

In one study, 13 patients with SRM who were either too old (median patient age was 69 years) or no candidates for surgery were followed with abdominal imaging for a median of 42 months. The growth rate was highly variable. Most SRM grew at a low rate or not at all [23]. In a subsequent study, 32 renal masses <4 cm (25 solid masses, 7 complex cysts) which were found in 29 patients were managed by surveillance. During a median followup of 27.9 months (range: 5.3–143.0 months) serial abdominal imaging was performed at least three times on each mass. The average growth rate was low and it did not show any statistical difference from zero growth (*P* = .09; 95% confidence interval, −0.005–0.2 cm per year) and was not associated with either initial size (*P* = .28) or mass type (*P* = .41). Seven masses (22%) reached 4 cm in greatest dimension after 12–85 months of followup. Eight masses (25%) doubled their volumes within 12 months. Overall, 11 masses (34%) fulfilled one of these two criteria of rapid growth. Nine tumors were removed surgically after an average of 3.1 years of followup either due to surgeon's concern or patient's anxiety. No progression to metastatic disease was observed [24].

There are a certain number of other retrospective studies investigating the natural history of SRM. Commonly these authors render surveillance as a possible safe option for patients with short-life expectancies or patients who are unfit for surgery. Unfortunately these studies accrued a limited number of patients and had short followup durations [25–29]. In their review, Rendon and Jewett [30] conclude active surveillance as a viable and safe option for the patient with a short-life expectancy or within well-controlled clinical trials. They also point out the necessity of close interval followups using imaging techniques every 3 months for the 1st year, every 6 months for the next 2 years and once every year thereafter.

In a meta-analysis by Chawla et al. [8], 234 SRM under surveillance were included. Mean lesion size at presentation was 2.60 cm (median 2.48, ranging from 1.73 to 4.08). Lesions were observed for a mean followup of 34 months (median 32, ranging from 26 to 39 in all series combined). The mean growth rate was 0.28 cm per year (median 0.28, ranging from 0.09 to 0.86) and only 1% of the patients developed metastatic disease. In 46% of the cases (131 out of 286), a pathological confirmation was available, which showed RCC in 92% (120 of 131). Among RCC, a mean growth rate of 0.40 cm yearly (median 0.35, ranging from 0.42 to 1.6) was observed. Lesion size at presentation did not correlate with growth rate (*P* = .46). Serial radiographic data alone were insufficient to predict the true natural history of SRM and patients concomitant diseases should also be taken into consideration when deciding for active surveillance.

Kouba et al. [31] reported short-term outcomes of patients under surveillance. A total of 43 patients with 46 renal masses underwent planned expectant management of enhancing solid or cystic (Bosniak IV) renal masses. 74% of patients had tumor growth with a mean (median) growth rate of 0.70 (0.35) cm per year during a mean followup of 36 months. There were no significant symptoms, disease progression or cancer-specific death. Four patients (10%) died of other causes. 13 out of 43 patients underwent surgical intervention after a mean delay of 12 months. Initial tumor size showed no significant difference in the intervention and nonintervention group (3.1 cm 2.6 cm, resp., *P* = .4504) and there was also no correlation between growth rate and tumor size. Delayed intervention did not appear to adversely impact pathological outcomes. The authors consider surveillance for SRM as a reasonable option for appropriately selected patients, especially the elderly and those with competing co-morbidities.

These data suggest that active surveillance is an option in elderly patients with severe comorbidities or patients, who are not willing to undergo surgery. Excellent patient compliance and close followup with contrast enhanced CT or MRI is mandatory.

### 5.2. Limitations

Imaging alone is inadequate on defining management in patients with SRMs. Punnen et al. observed inter- and intraobserver variability in measuring tumor diameter (±0.3 cm in diameter). Tumor volume is exponentially related to tumor diameter, and thus inaccuracy of measuring tumor diameters is related to a greater error (inter- and intraobserver variability for tumor volume 2.515 mm^3^ and 2.075 mm^3^, resp.) [32].

### 5.3. Role of renal tumor biopsy

There have been serious suspicions in the past about needle-core biopsies of renal masses regarding complications, tumor seeding, and wrong sampling. Due to advances in application techniques and help of imaging guidance, the results of needle biopsies have improved significantly. Fine-needle aspiration (18-gauge or thinner) and core biopsies of renal masses are now much safer than before and they can even be applied in an outpatient setting with low morbidity rates [33–36].

Today the success rate of obtaining tissue and their pathologic interpretation are excellent [37]. Neuzillet et al. [34] were able to obtain adequate material for histological examination from 96.6% out of 88 patients that underwent Helical-CT-guided percutaneous fine-needle biopsy. 62 patients whose biopsy examinations indicated RCCs were treated surgically (radical or partial nephrectomy). The postoperative evaluation revealed a 92%-sensitivity rate of biopsy in predicting malignancy and tumor subtype. The results also showed no false-positive cases, no track seeding, and no complications.

Schmidbauer et al. [37] published a prospective study on 78 patients with SRMs who underwent 18-gauge core biopsy under computed tomography (CT)-fluoroscopic guidance. In addition, using the same sheath, fine-needle aspiration was taken in 44 patients and analyzed cytologically. The renal masses were subsequently removed surgically and evaluated histologically. The results showed a sensitivity of 93.5% and 90.6%, for core biopsy and fine-needle aspiration for the detection of renal cell carcinoma (RCC), respectively; Fuhrman grade was correctly predicted in 76% and 28% and the correct histologic subtype identified 91% and 86%, respectively. Cytology from fine-needle aspiration revealed a sensitivity of 100% and 75% in detecting malignant and benign lesions, respectively. Two of the SRMs' diagnosed as oncocytomas on core biopsy were revealed to be hybrid tumors with scattered areas of oncocytomas and chromophobe RCC on histological evaluation.

These data suggest that before opting for surveillance a sufficient renal tumor mass biopsy should be performed to further guide followup.

## 6. WHO AND HOW?

According to many authors, active surveillance is a feasible option especially for elderly and unfit patients. Because active surveillance is a new concept, large studies with appropriate followup are still missing. Which patients should undergo active surveillance? What should be our followup intervals? What is the cut-off tumor diameter? What should be the limit for annual growth? In order to precisely answer these questions, larger numbers of studies are required. In some centers, renal masses below the limit of 3-4 cm in diameter are considered to be at low risk of metastasis [38, 39].

The authors believe that low-grade tumors measuring <3 cm could enter an active surveillance protocol. Prior and during followup, renal tumor biopsies are mandatory. Biopsies should be assessed by the same pathologist to exclude interobserver variations, especially in grading. High-grade tumors, sarcomatoid features, collecting duct, and unclassified RCC have to be excluded because of their known unfavorable outcomes. Additionally, young and/or healthy patients are no good candidates for active surveillance because of lacking long-term data (see Table 1).

For followup, Rendon et al. suggested CT or MRI every 3 months in the 1st year, every 6 months for the next 2 years and every year thereafter [30], however, there are no oncological outcome data that support this approach. Again, repeated biopsies of the mass have to be performed in certain intervals. Tumors that exceed 3 or 4 cm or which double in volume in <12 months need further intervention [7, 10, 11], for example, surgery or ablation.

These short-interval followups may have a negative impact on patient compliance. Another downside of active surveillance is the patient's anxiety. The knowledge of living with a tumor and “not doing anything about it” is also a psychological burden on the patient.

## 7. CONCLUSIONS

Active surveillance is a new management option for the aged and infirm patient with short-life expectancy. The current literature contains mostly small, retrospective studies with limited data. Prior and during followup, renal tumor biopsies are mandatory. Thus, the authors rather believe in active surveillance than in classical observation. Imaging alone is inadequate for suggesting the aggressive potential of SRM for both diagnosis and followup. Current data suggest that a CT or MRI every 3 months in the 1st year, every 6 months in the next 2 years and every year thereafter, is appropriate for observation.

Since not all SRM are harmless, selection criteria for active surveillance need to be improved. In addition, there is need for larger studies in order to better outline oncological outcome and followup protocols.

## Figures and Tables

**Table 1:** Possible inclusion and exclusion criteria for active surveillance of SRM.

*Inclusion:*

(1) Benign lesion on renal tumor biopsy
(2) Aged and infirm patient
(3) Tumor size <3 cm on cross-sectional imaging
(4) Chromophobe RCC, low-grade, on renal tumor biopsy
(5) Chromophobe-oncocytic hybrid tumor on renal tumor biopsy
(6) Willingness of the patient to undergo regular CT or MRI scans and repeated biopsies (good compliance)

*Exclusion:*

(1) Young and healthy patient
(2) Sarcomatoid features
(3) Collecting duct or unclassified RCC
(4) Evidence of ≥T3a disease on cross-sectional imaging
(5) High grade
(6) Symptomatic lesions

*Unclear:*

(1) Low grade clear-cell RCC
(2) Low-grade papillary RCC
(3) Multifocal RCC
(4) Hereditary RCC
